# Humanized CD30-Targeted Chimeric Antigen Receptor T Cells Exhibit Potent Preclinical Activity Against Hodgkin’s Lymphoma Cells

**DOI:** 10.3389/fcell.2021.775599

**Published:** 2022-01-12

**Authors:** Jing Guo, Shuai He, Yongjie Zhu, Wei Yu, Dong Yang, Xudong Zhao

**Affiliations:** Laboratory of Animal Tumor Models, Frontiers Science Center for Disease-related Molecular Network, West China Hospital, Sichuan University, Chengdu, China

**Keywords:** chimeric antigen receptor, CD30, humanized HRS3 antibody, single-chain variable fragment, Hodgkin’s Lymphoma, central memory phenotype

## Abstract

CD30-directed chimeric antigen receptors (CARs) with single chain antibody fragment (scFv)-binding domains from murine HRS3 show strong cytotoxicity to Hodgkin’s Lymphoma cells and have been used in clinical trials. However, murine scFv in CAR might induce specific rejective immune responses in patients, which compromises the therapeutic effects. The use of human or humanized antibody fragments for CAR construction, rather than those derived from mouse antibodies, can reduce the immunogenicity of the CAR. Importantly, this strategy might simultaneously decrease the risk of cytokine-mediated toxicities and improve CAR T cell persistence. Murine HRS3 antibody has been successfully humanized by grafting the complementarity-determining regions (CDRs) from the mouse antibody framework onto human immunoglobulin consensus sequences, followed by an *in vitro* evolutionary strategy to select functional Fab fragments with the same affinity as murine sources. In this study, humanized scFvs were utilized to construct a CD30-directed CAR (hHRS3-CAR), and its effectiveness was compared with that of HRS3-CAR. The hHRS3-CAR-T cells specifically kill CD30-positive tumor cell lines *in vitro* and eliminate lymphoma xenografts in immunodeficient mice with comparable efficiency to HRS3-CAR. The hHRS-CAR-T could be used in clinical trials based on the previously reported advantages of humanized CARs, such as the reduction of immune rejection and better persistence of cells.

## Highlights

• hHRS3-CAR-T cells were highly efficacious against CD30-positive tumor cells similar to HRS3-CAR-T cells
• hHRS3-CAR-T cells show higher central memory phenotype compared to HRS3-CAR-T cells
• The CD30-directed CAR-T based on humanized HRS3 scFv might decrease immunogenicity and benefit the clinical therapy

## Introduction

Chimeric antigen receptors (CARs) are artificial proteins whose basic structure is composed of an antigen recognition ectodomain and activation endodomain linked by a spacer and transmembrane domain (Eshhar et al., 1993; Sadelain et al., 2013; Boyiadzis et al., 2016). These are engineered receptors used to specifically direct patient T cells to target tumor cells (Wang et al., 2019). Adoptive cell therapy with the cluster of differentiation 19 (CD19)-directed chimeric antigen receptor T-cells (CAR-T) has induced durable clinical efficacy and has been approved for clinical first-line treatment, which provides a rationale for the development of other CARs (Kochenderfer et al., 2012; Boyiadzis et al., 2016; Turtle et al., 2016b).

Tumor necrosis factor receptor superfamily member 8 (TNFRSF8) is a 120-kDa type I transmembrane glycoprotein, more commonly referred to as CD30 (van der Weyden et al., 2017). In the normal physiological state, cell surface expression of CD30 is limited to activated T, B, and natural killer lymphocytes (Wasik et al., 2013). However, it is also strongly expressed in malignant hematopoietic cells, including Hodgkin’s lymphoma (HL), anaplastic large cell lymphoma (ALCL), primary cutaneous ALCL, lymphomatoid papulosis, and certain cases of transformed mycosis fungoides (Schirrmann et al., 2014; van der Weyden et al., 2017). Thus, it was regarded as an ideal therapeutic target and a wide range of agents were developed for CD30-positive malignancies (Schwarting et al., 1989; Younes et al., 2012). Brentuximab vedotin, a CD30 antibody–drug conjugates, has achieved remarkable results against HL and ALCL (Pro et al., 2012; Younes et al., 2012). Recently, it was shown that anti-CD30 CAR-T cells can induce partial or complete remission in HL and ALCL (Wang et al., 2017; Ramos et al., 2020).

Although CD30 remains an excellent target for CAR-T therapy of HL, various obstacles should be overcome to clinically improve their efficacy, such as the persistence of CAR-T cells, trafficking to tumors, and increased tumor cytotoxicity (Ramos et al., 2017; Hong et al., 2018; Grover and Savoldo, 2019; Hombach et al., 2019). The murine anti-CD30 monoclonal antibody (HRS3), and HRS3-based bispecific antibody (AFM13) and recombinant fusion protein combined with cytokines, and CAR-T have been developed and applied to the specific immunotherapy of Hodgkin’s lymphoma, including CAR-T therapy (Hombach et al., 1993; Renner et al., 2000; Jahn et al., 2012; Grover and Savoldo, 2019). Currently, most single chain antibody fragments (scFvs) are murine-derived, which, owing to immunogenicity, may weaken the efficacy of the treatment (Zou et al., 2018). One strategy to reduce the immunogenicity of CAR-T cells is to humanize scFv. Humanized CAR-T cells have also received attention because they are less antigenic and have a long survival time *in vivo* (Turtle et al., 2016a; Sommermeyer et al., 2017; Rafiq et al., 2020). Starting from the previously characterized cognate HRS3 mouse monoclonal antibody, the bacterially produced functional Fab fragment was humanized by grafting the CDRs from the mouse antibody framework onto human immunoglobulin consensus sequences, followed by an *in vitro* evolutionary strategy to select functional Fab fragments with the same affinity as murine sources (Schlapschy et al., 2004). The humanized HRS3 antibody is fully functional in recognizing its native receptor antigen. CAR-T based on humanized HRS3-scFv has not been reported.

In this study, murine and humanized HRS3 scFv-based CARs (HRS3-CAR and hHRS3-CAR, respectively) were constructed. We used humanized scFvs to construct hHRS3-CARs, and its effectiveness was compared with that of HRS3-CAR.

## Materials and Methods

### Cell Culture

L428 and L540 Hodgkin’s lymphoma-derived cell lines were purchased from Shanghai Fudan-Zhangjiang Bio-Pharmaceutical Co., Ltd. (Shanghai, China). Cells were cultured in RPMI-1640 (Hyclone, United States) supplemented with 20% fetal bovine serum (FBS; Gibco). Raji cells were purchased from ATCC and cultured in RPMI-1640 medium containing 10% FBS. HEK293T cells were cultured in Dulbecco’s modified Eagle’s medium (Millipore, United States) supplemented with 10% FBS at 37°C and 5% CO_2_. The cells were authenticated by via Short Tandem Repeat (STR) profiling and tested negative for *mycoplasma* contamination.

### Construction of Plasmids Encoding anti-CD30 CARs

The CD19-specific single-chain antibody CD19-scFv was derived from anti-CD19 mAb as previously described (Yang et al., 2017). The VL and VH regions of murine and humanized HRS3 and HRS3 antibodies were obtained from the published article (Schlapschy et al., 2004). The extracellular domains containing VL and VH regions were synthesized and cloned into a lentiviral backbone containing other parts of CAR: a CD8 hinge spacer and transmembrane region, 4-1BB, and CD3ζ endo-domains under the control of the CMV promoter. The resulting plasmids were named HRS3-CAR and hHRS3-CAR, respectively.

### Lentiviral Preparation and Transduction of T-Cells

Lentiviral DNA vectors were transfected with Lipo6000™ Transfection Reagent (Beyotime, China) according to the manufacturer’s protocol. For lentivirus production, 20 μg of core plasmid together with helper plasmids (10 μg pCMVΔ8.9 and 4 μg pMD2. G) were transfected into 293T cells as described previously (Yang et al., 2018). Viral supernatants were collected 48 and 72 h after transfection and filtered through a 0.45 μm filter. After centrifugation at 25,000 rpm for 2.5 h at 4°C, the virus was suspended in 0.1% bovine serum albumin (BSA) in PBS, dissolved overnight and stored in aliquots at 80°C.

Primary human T cells were isolated from healthy human blood, as previously described (Yang et al., 2019). T cells were cultured in advanced RPMI 1640 medium (Life Technologies, United States) containing 10% FBS (Life Technologies, United States) with 200 U/ml IL-2 (PeproTech, United States). T cells were activated by adding Dynabeads human T-activator CD3/CD28 kit (Life Technologies, United States) according to the manufacturer’s instructions. After 48 h, at a 1:1 ratio, and on day 3, T cells were transduced with lentivirus (Multiplicity of infection, MOI = 20) in the presence of Lentiboost (Sirion Biotech), followed by a medium change after 24 h transfer to 24-well plates.

### Cytotoxicity Assay

Cytotoxicity assays were conducted using a slightly modified version of a previously described assay, and cytokine concentrations were determined using enzyme-linked immunosorbent assay (ELISA) (Sun et al., 2019). For data analysis, target cells were labeled with carboxyfluorescein succinimidyl ester (CFSE) and dead target cells were identified as double-positive for CFSE and propidium iodide (PI) using a FACS Canto flow cytometer. For each sample, 10000 events of P1 were collected. The percentage of specific target cell death was calculated as follows: 100 × [No. of CFSE and PI double-positive cells] ÷ [(No. for CFSE-positive cells) + (No. of CFSE and PI double-positive cells)].

### Flow Cytometry

Flow cytometry was performed using the standard methods (Yang et al., 2019). The following antibodies were used: PE-anti-CD3 (Clone UCHT1, BD Biosciences or Biolegend, 981002), APC-anti-CD4 (BioLegend, 357408), FITC-anti-CD8 (BioLegend, 344704), PE-anti-CD45RO (Biolegend, 304206), APC-anti-CCR7 (BioLegend, 353214), and CD30 APC (Clone BerH8, BD Biosciences). Dead target cells were identified by double staining with CFSE and PI (BD Biosciences). All experiments were analyzed using FlowJo version 10 (Tree Star, Inc.).

### Xenograft Mouse Model

The protocol was approved by the Ethics Committee of the West China Hospital of Sichuan University West China Hospital. Six-to eight-week-old female NOD-Prkdcem26ll2rgem26Nju (NCG) mice were purchased from GemPharmatech Co., Ltd (Jiangsu, China) and engrafted with 2 × 10^5^ L428-EGFP-luci cells via tail vein injection. Five days later, CAR- or control T cells that had been expanded *in vitro* for 7 days were injected intravenously. The mice were serially imaged using 2D bioluminescence imaging as previously described to determine tumor progression (Guercio et al., 2021). All captured images were analyzed using Living Image software 4.1 (PerkinElmer, United States). The animals were examined every day for survival and sacrificed when moribund for collection of tissue samples. At the end of the experiment (day 64), the remaining surviving mice were euthanized and animal samples were collected. Each experiment included 6 mice per group and was repeated twice (total *n* = 12 mice per group).

### Cytokine Secretion Assays

Effector cells (2.5 × 10^5^ NC, CD19-CAR, HRS3-CAR, or hHRS-CAR-T cells) and target cells (5 × 10^4^) were co-cultured in the absence of IL-2 at a ratio of 5:1 in 96-well plates for 24 h. Supernatants were collected and subjected to ELISA assay (BD Biosciences, United States) measurements according to the manufacturer’s instructions.

### Statistical Analysis

Graphs were plotted using the GraphPad Prism 8.0 statistical software. Data were analyzed using SPSS 22.0 software (IBM, United States). Statistical differences between two groups were analyzed using the unpaired Student’s t-test with Welch correction. Unless otherwise stated, data are presented as mean ± SD, and statistical significance was set at *p* < 0.05.

## Results

### Humanized HRS3-CAR-T Cells Exert a Similar *In vitro* Anti-Lymphoma Activity Compared to HRS3-BBz T-Cells

**FIGURE 1:**
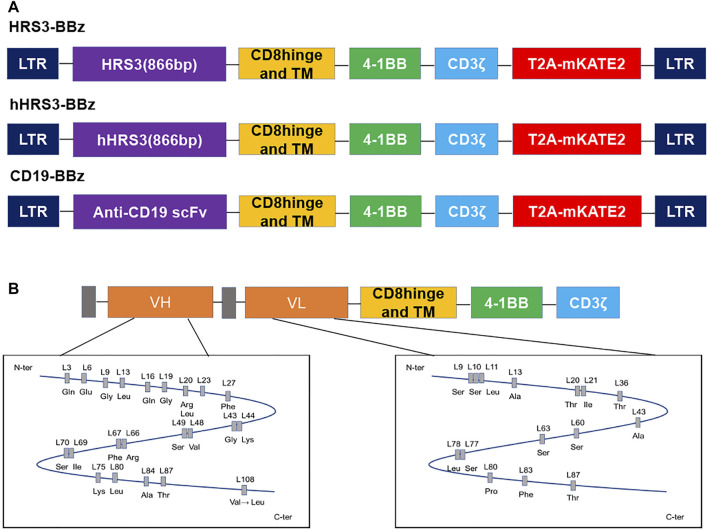
Schematic diagram of the humanized HRS3-chimeric antigen receptor (CAR) construct and its differences from the murine receptor. **(A)** Schematic representation of CD19-CAR, HRS3-CAR, and hHRS3-CAR. All CARs had hinge, transmembrane, and CD8, and a CD3ζ T-cell activation domain. The mKATE2 is far-red fluorescent protein. In this study, the mKATE2 domain was used to trace the expression of CAR by fusing to the N-terminal of CAR via a self-cleaving T2A peptide. **(B)** Humanization of the HRS3 VL domain required 18 amino acid exchanges while retaining one amino acid from the murine framework sequence, whereas for the HRS3 VH domain, 27 amino acids were exchanged and four murine residues were retained (Schlapschy et al., 2004). The figure shows the schematic diagram of amino acids after mutation.

We constructed hHRS3-CAR and HRS3-CAR for an initial validation of the humanized CAR, by cloning the scFvs of hHRS3 or HRS3, respectively, into the same CAR scaffold (Figure 1A) (Schlapschy et al., 2004). The CAR expressing anti-CD30(HRS3)-scFv was used as the positive control and that expressing anti-CD19-scFv was used as the negative control (CD19-CAR), as previously described (Sun et al., 2019). Comparing the amino acid sequences of VL and VH for HRS3-scFv and hHRS3-scFv, it was found that there were no significant changes in the amino acids in the VL; however, amino acid changes in the VH were apparent (Figure 1B).

**FIGURE 2:**
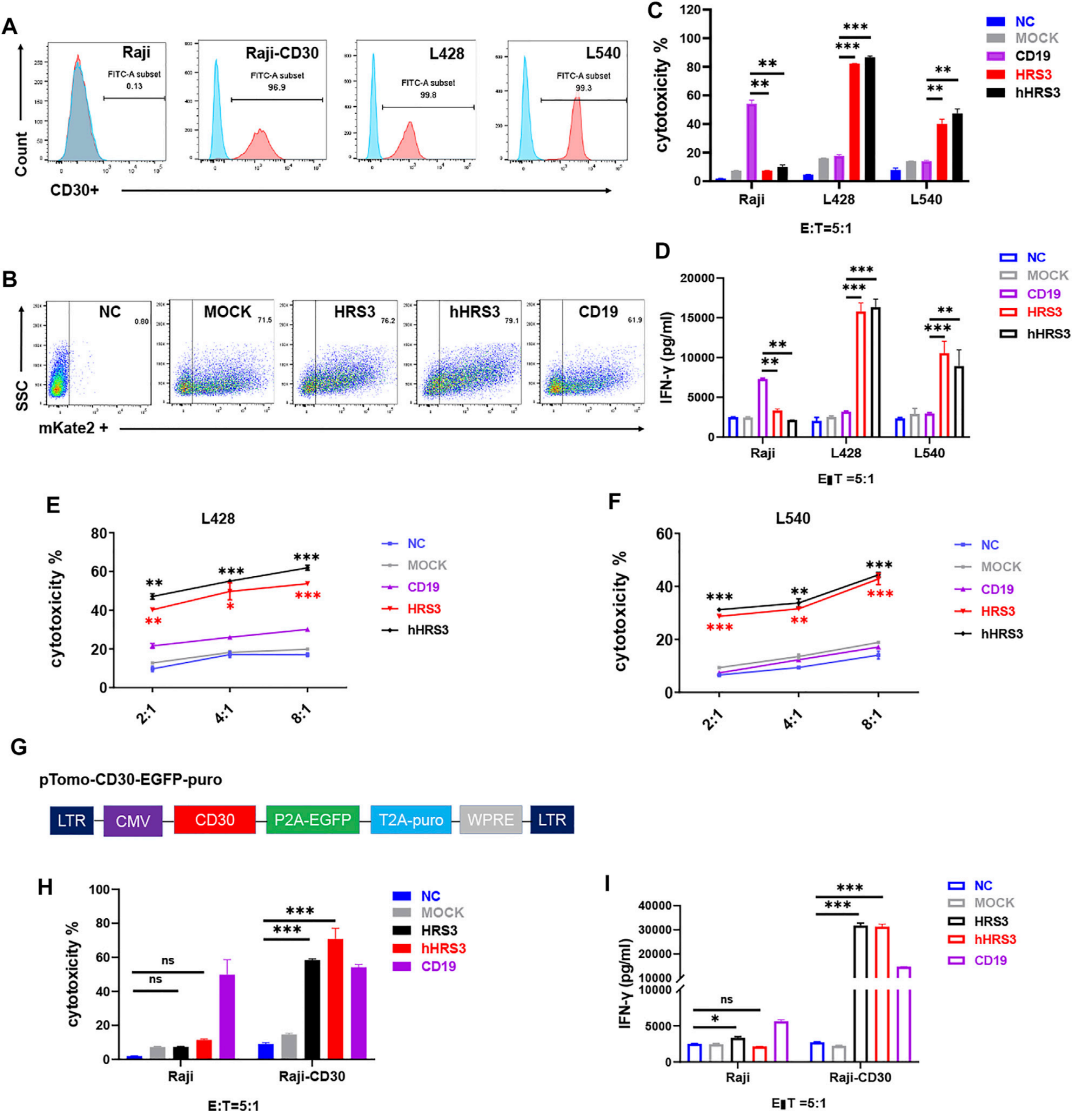
CARs with humanized HRS3-specific scFv recognize CD30^+^ tumor cells. **(A)** B cell lymphoma and Hodgkin’s lymphoma (HL) cell lines were stained with a monoclonal antibody specific for the CD30 antigen and analyzed using flow cytometry. CD30 expression was evident in a majority of the cell lines after staining with an anti-CD30 monoclonal antibody (Pink) when compared to staining with control antibody (Blue). Raji was used as negative control. **(B)** Flow-cytometry analyses in a representative donor showing CAR expression. **(C)** T cells expressing hHRS3-CAR and HRS3-CAR exhibited cytotoxicity against the non-HL and HL cell lines in a 24-h assay. Graphs show mean ± SEM of duplicate wells. One of two representative experiments with different donor cells and similar results is shown. **(D)** IFN-γ concentrations in the supernatants of CD30-CAR-T-cells after stimulation with Raji, L428, and L540 cells for 24 h, analyzed by ELISA. **(E,F)** *In vitro* cytotoxic assay to evaluate cytolytic activity of NC T cells (Blue line), CD19-CAR-T cells (Purple line), HRS3-CAR-T cells (Red line) and hHRS3-CAR-T cells (Black line) on CD30^+^ lymphoma cell lines, namely L428 and L540 cells. The results are presented as the mean volume ±SD, * p-value < 0.05, ** p-value < 0.01, *** p-value < 0.001 vs CD19 (CD19 as negative control). **(G)** Diagram of the full-length overexpressing human CD30 vector. **(H,I)** Detection of Raji and Raji-CD30 cell killing and cytokines (CD19 as positive control). “NC” represents uninfected T cells (negative control). “MOCK” indicates the transgenic plant that contained only the vector construct (negative control). The data were derived from different donor cells and the *in vitro* experiments were repeated independently at least three times. The results are presented as the mean volume ±SD, * p-value < 0.05, ** p-value < 0.01, *** p-value < 0.001 vs NC.

To verify the function of hHRS-CAR-T, we selected non-HL cells Raji and U937, and the HL cells L428 and L540. We evaluated CD30 protein expression using flow cytometry. CD30 was not detected in Raji or U937 cells, but was detected in the HL cell lines (Figure 2A, Supplementary Figure S1A). Next, we examined CAR expression and evaluated the ability of CAR-T cells to mediate the lysis of CD30^+^ HL cells (Figure 2B). CAR-T cells lysed CD30^+^ L428 and L540 cells (Figure 2C), but not Raji and U937 cells (Supplementary Figure S1B). We assessed the cytotoxicity of CD30-CAR T cells using 24-h cytotoxicity assays (Figure 2D). T cells expressing CD19-CAR served as negative controls. CAR-T cells specifically recognized and eliminated HL cells in an effector-to-target ratio-dependent manner (Figures 2E,F).

### Humanized HRS3-CAR-T Specifically Kills CD30-Positive Cells Similar to HRS3-CAR-T

To test whether T cells expressing hHRS3-CAR were capable of specifically recognizing tumor lines expressing CD30, we co-incubated CAR-T cells with Raji cells expressing CD30 and those that did not (Figure 2G). CAR-T cells specifically lysed Raji-CD30 cells, but not Raji cells (Figure 2H). The amounts of secreted cytokines, IFN-γ and IL-2 (Figure 2I, Supplementary Figure S2), were measured. The hHRS3-CAR-T cells recognized all CD30^+^ tumor lines and secreted high levels of IFN-γ. Very low levels of IFN-γ were observed when hHRS3-CAR-T cells were co-cultured with Raji cells. In contrast, higher levels of IFN-γ were observed when CD19-CAR-T cells were co-cultured with Raji cells.

### Expanded hHRS3-CAR-T Cells Express a Higher Central Memory Phenotype

**FIGURE 3:**
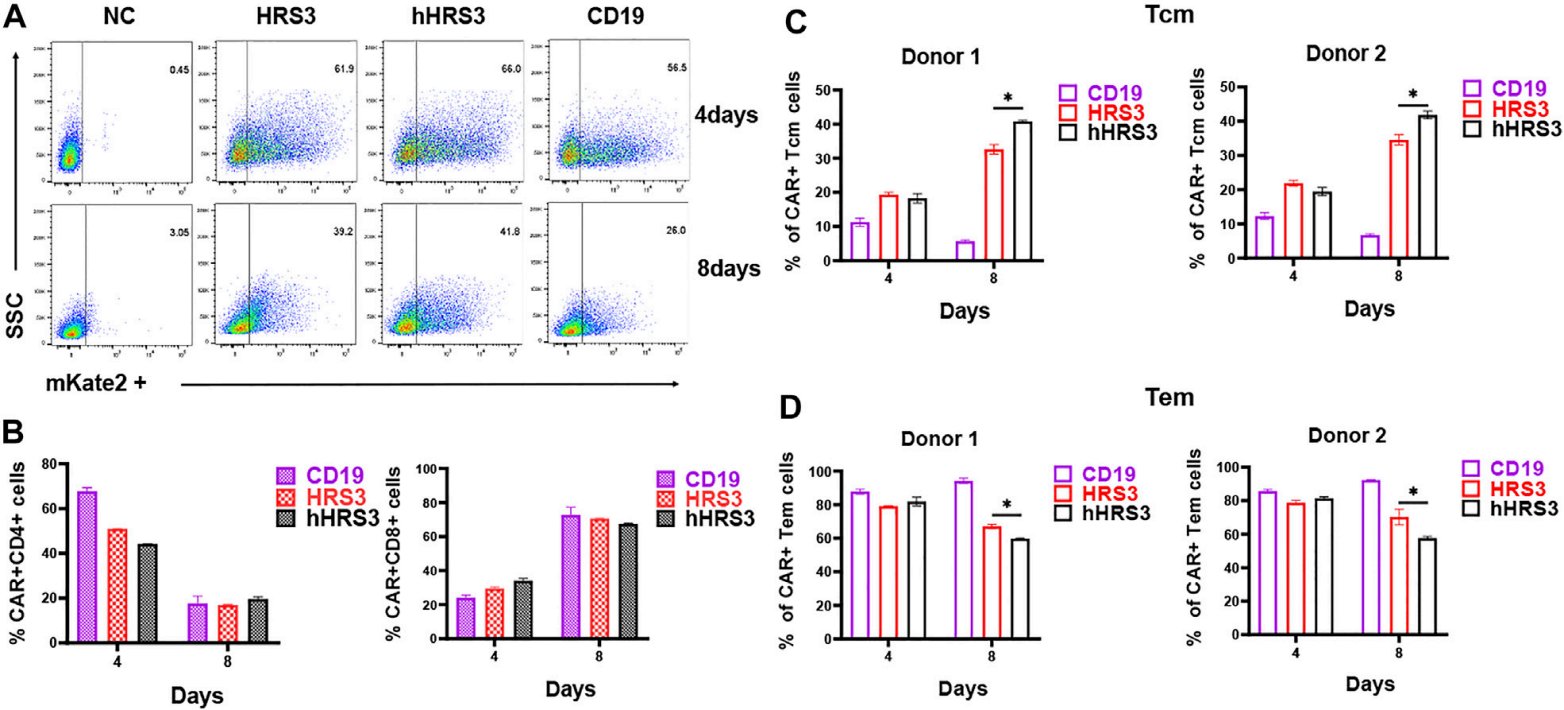
Detection of CAR-T cell phenotypes. **(A)** Three days after culture initiation, the cells were transduced with vectors encoding the indicated CARs, or the cells were left untransduced. Cells were also stained with Protein L to detect CARs. **(B)** Four and 8 days after transduction, the cells were stained with antibodies against CD4, and CD8. There were no significant differences between T cell expression for each CAR on day 4 and 8 post-infection. **(C)** T cell subpopulations were analysed in different CARs by flow cytometry (mean ± SD). T cells are functionally divided into four subsets: central memory (CCR7^+^CD45RO^+^) and effector memory (CCR7^−^CD45RO^+^), according to the cell surface expression of CCR7 and CD45RO. The *in vitro* experiments were independently repeated at least three times using different donor cell sources in figure A, B. The data in Figure **(C,D)** were from two independent experiments with three wells repeated per experiment. The results are presented as the mean volume ±SD, * p-value < 0.05 vs HRS3.

To identify the effect of CAR expression on T cell phenotypes, markers of the T cell subsets were analyzed using FACS. The CD4 to CD8 ratio of CAR^+^ T cells was not significantly different between the HRS3-CAR-T and hHRS3-CAR-T cells (Figures 3A,B). Notably, participating effector memory (Tem) cells were significantly enriched in CD30-CAR-T cells 4 days after infection, whereas central memory (Tcm) cells were significantly enriched in CAR-T cells on the eighth day after infection (Figures 3C,D). The hHRS3-CAR-T cells maintained a higher proportion of CCR7^+^CD45RO^+^ Tcm cells, while HRS3-CAR-T cells had larger populations of CCR7^−^CD45RO^+^ Tem cells compared to their counterparts.

### Antitumor Responses by Humanized HRS3 Chimeric Antigen Receptor T Cells *in vivo*

**FIGURE 4:**
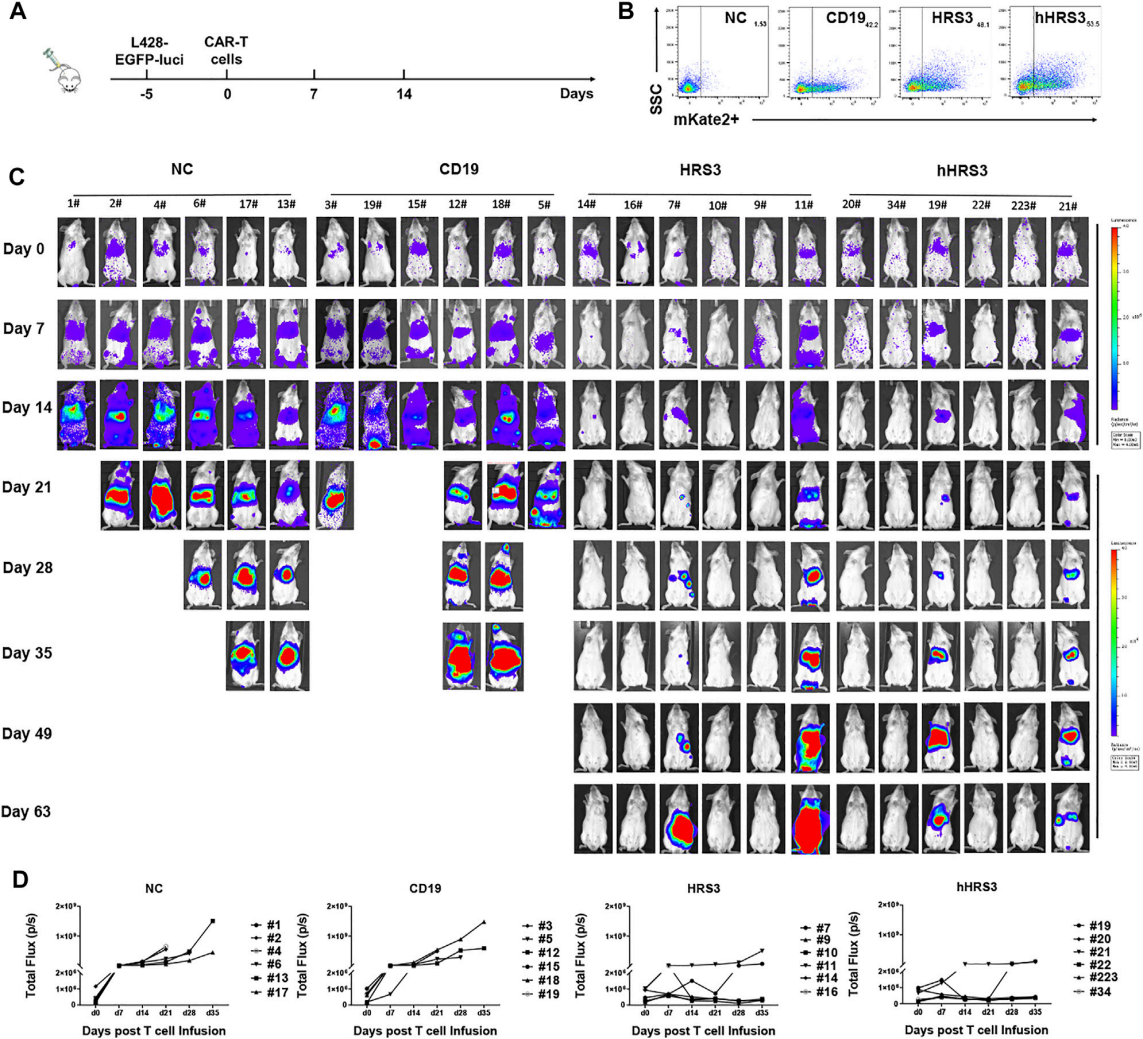
Humanized HRS3-CAR-T cells eradicate tumors *in vivo*. **(A)** The panel shows the *in vivo* xenograft immunodeficient mouse model in which HL L428-EGFP-luci cells (2 × 10^6^ cells per mouse) were systemically infused into NCG mice (*n* = 6 for both experimental and control groups, day -5). Effector cells or un-transduced T cells (1 × 10^7^ cells per mouse) were infused intravenously at the time of tumor establishment (day 0), which was assessed by IVIS Imaging. **(B)** Expression of CAR-T in each group inoculated. **(C)** IVIS Imaging of tumor growth from day 0 to day 63 (end-of-imaging). **(D)** Bioluminescence of each mouse xenograft treated with NC T cells, CD19-CAR-T cells, HRS3-CAR-T cells, and hHRS3-CAR-T cells. Each experiment included 6 mice per group and was repeated twice (total *n* = 12 mice per group). Data are representative of two independent experiments.

**FIGURE 5:**
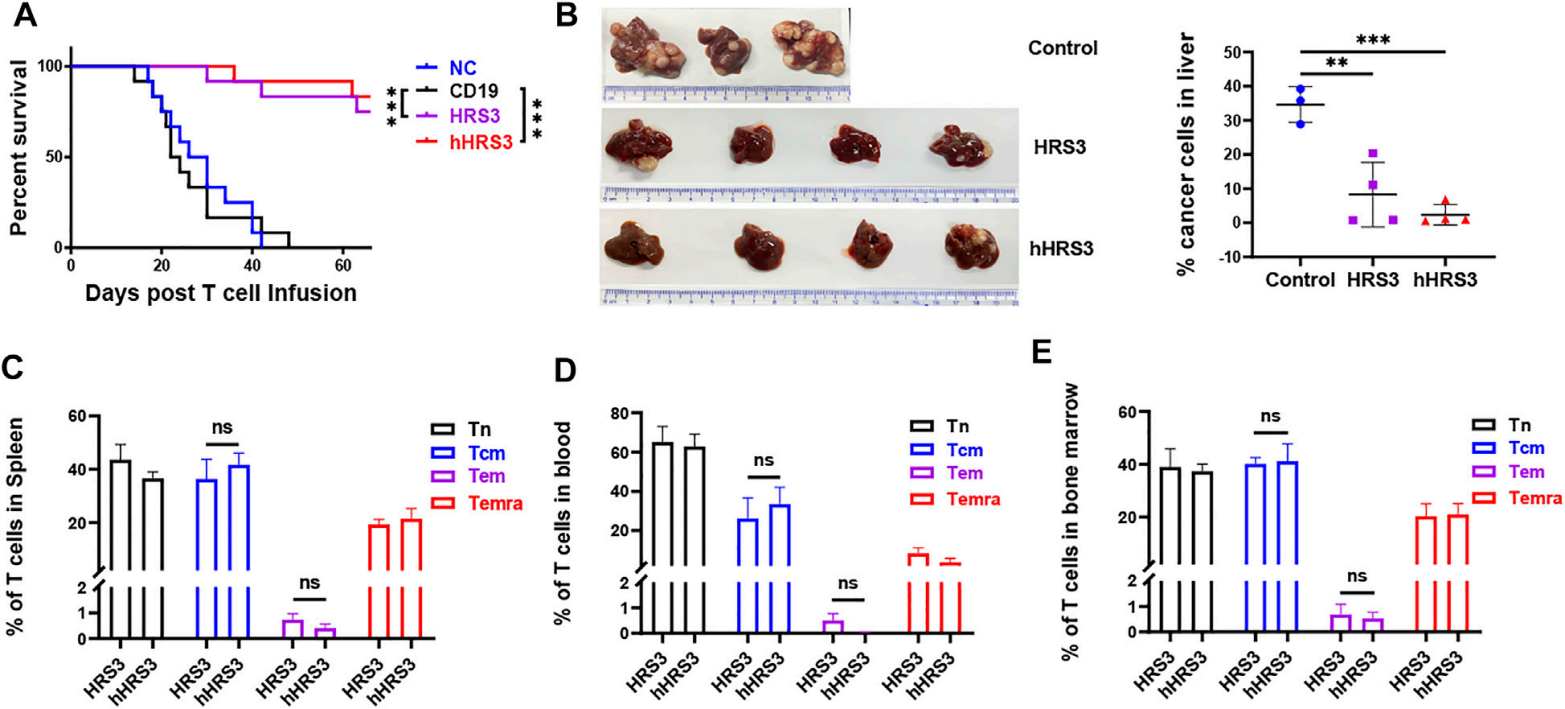
Central memory cells (Tcm) expressing hHRS3-CAR were enriched in mice and conferred long-lasting immunity to HL. **(A)** Survival of mice is shown as a Kaplan-Meier curve (data shown combined from two independent experiments, *n* = 12/group). Survival was not statistically different for NC mice versus CD19; *p* = 0.8037. *p* < 0.0001 for the comparison of HRS3 group versus CD19 mice. *p* < 0.0001 for the comparison of hHRS3 group versus CD19 mice. NC, non-transduced T cells. **(B)** Flow cytometry detection of tumor cell involvement in the liver of mice in each group. The control group was CD19 group (*n* = 3), and the experimental group was HRS3 and hHRS3 groups (*n* = 4). **(C)** Spleen, **(D)** blood, and **(E)** bone marrow from surviving CD30-CAR-treated mice (*n* = 3) were analysed for the number of CAR-T cell subsets using flow cytometry (mean ± SD). T cells are functionally divided into four subsets: naïve (CCR7^+^CD45RO^−^); central memory (CCR7^+^CD45RO^+^); effector memory (CCR7^−^CD45RO^+^); and terminal effector (CCR7^−^CD45RO^−^), according to the cell surface expression of CCR7 and CD45RO. Each experiment included 6 mice per group and was repeated twice (total *n* = 12 mice per group). **(B)** data shown are representative or **(C–E)** combined from two independent experiments (*n* = 12). * p-value < 0.05, ** p-value < 0.01, *** p-value < 0.001 vs control (CD19).

To evaluate the anti-tumor activity of hHRS3-CAR-T and HRS3-CAR-T cells in murine models, NCG mice (six per group) were injected intravenously with L428 tumor cells (2 × 10^6^ cells per mouse). After 5 days (day 0), the tumor-bearing mice were imaged using a bioluminescence imaging system for Luc expression, and the mice were randomly divided into four groups for different treatments (Figures 4A,B). NC T cells, CD19-CAR-T cells, HRS3-CAR-T cells, and hHRS3-CAR-T cells (1 × 10^7^ cells per mouse) were infused intravenously as a single dose. hHRS3-CAR-T cells showed excellent efficacy comparable to HRS3-CAR cells. In contrast, CD19-CAR-T and NC T cells exhibited progressive tumor growth (Figure 4C). When tumor sizes were assessed 21 days after CAR-T cell infusion, we found that T cells expressing hHRS3-CAR were more effective at reducing tumor sizes than T cells expressing HRS3-CAR (Figure 4D); however, survival and weight change were not statistically different when treatment with hHRS3-CAR-T and HRS3-CAR-T cells was compared. Mice in the control group died after 40 days, whereas mice in the HRS3-CAR-T and hHRS3-CAR-T groups died after 63 days. Similar relative survival curves were obtained in repeated experiments, and the survival curve data from both experiments were combined for analysis (Figure 5A). Owing to significant liver ascites and death in the control group, body weight changes were only recorded for 35 days (Supplementary Figure S3). Previous studies have reported a class of HL tumor models that preferentially caused accumulation of tumors in the liver abdomen (Guercio et al., 2021). Macroscopic analysis of the sacrificed mice showed large tumor masses located preferentially in the liver (Figure 5B). Therefore, the anti-tumor activity of hHRS3-CAR-T cells was comparable to that of HRS3-CAR-T cells in this model.

Considering that hHRS3-CAR Tcm cells were significantly enriched *in vitro*, we evaluated its proportion *in vivo*. The results showed that the difference in Tcm cell ratio was not statistically significant in the blood, spleen, and bone marrow of mice treated with hHRS3-CAR-T cells versus HRS3-CAR (Figures 5C–E).

## Discussion

To date, several clinical trials have been conducted in adults with relapsed/refractory CD30^+^ lymphoma using different CD30-CAR-T cells (Di Stasi et al., 2009; Wang et al., 2017). Compared with the great success achieved by CD19-redirected CAR, CD30-target CAR still has room for improvement. Previous studies have demonstrated that the transfer of autologous human T-cells expressing foreign proteins, including CARs derived from murine scFvs, can elicit cellular and humoral immune responses that may compromise CAR-T effects (Lamers et al., 2006; Wang et al., 2020; Wagner et al., 2021). The use of human or humanized antibody fragments for CAR construction, rather than those derived from mouse antibodies, such as the production of modified CD19 and Her-2 CAR-T, combination of humanized or fully human fragments, and modification of the extracellular hinge region and/or transmembrane domain, can reduce the immunogenicity of the CAR (Hombach et al., 2010; Jonnalagadda et al., 2015). Importantly, this strategy might simultaneously decrease the risk of cytokine-mediated toxicity and improve CAR-T cell persistence (Sommermeyer et al., 2017; Rafiq et al., 2020).

HRS3 is derived from a murine antibody (da Costa et al., 1992; Hombach et al., 1998); humanized HRS3 scFv was derived from HRS3 mAb, which may represent another class of anti-CD30 antibodies (Schlapschy et al., 2004). Notably, previous studies have demonstrated that the humanized Fab fragment was fully functional with respect to CD30 binding; however, whether the CAR possessing this molecule as an extracellular recognition domain can safely and effectively eliminate target cells remains requires further investigation. In this study, we found that hHRS3-CAR-T cells were highly efficacious against antigen-specific tumor cells in both *in vitro* and *in vivo* with responses that were comparable to those of HRS3-CAR-T cells. Besides scFv, other elements of CAR may also produce rejection. In this study, the sequence of the hinge spacer and transmembrane domain in the CAR were all derived from human CD8α to minimize immunogenicity. Furthermore, the linker of (GGGGS)3 that connects the heavy and light chains in the scFv has been widely used in antibodies and CARs. However, these strategies do not completely eliminate immunogenicity. Even a fully human antibody fragment might also lead to immune reactions in the human; therefore, to conclusively determine whether they are nonimmunogenic requires their application in human clinical trials.

Retrospective analysis of published CAR-T cell clinical studies revealed that an elevated proportion of Tcm or less differentiated CAR-T cells provided superior antitumor efficacy, and Tcm-derived CAR-T cells are functionally superior to those made from bulk CD8^+^ T cells (Chang et al., 2015; Tao et al., 2020). Tcm provides effective long-term memory responses because they have the capacity to persist long term in the circulation, have a high proliferative capacity, and can replenish other memory T-cell subsets, including Tem (Gehad et al., 2018; Mauriello et al., 2020). Our results showed that hHRS3-CAR could produce the higher proportion of Tcm compared to parental CAR. Similar results were also observed in previous reports (Zhao et al., 2019). However, the mechanism underlying this effect remain to be elucidated up to now. We speculate that it is due to the continuous stimulation caused by antigen, such as murine scFv, accelerating the differentiation of Tcm to Tem. Although the advantages of humanized CAR-T have been proven in clinical trials, the improvement was not significant in our experiment, which may be due to the huge differences in the immune environment in immunodeficient mice and human. For example, the humanized anti-CD19 CAR did not show a difference in the ability to kill target cells *in vitro* and in mice compared to parental CAR, but it exhibited significantly lower side effects in the patients with B-cell lymphoma (Heard et al., 2021).

In conclusion, we focused on developing a CD30-specific CAR using humanized HRS3-scFv. hHRS3-CAR-T cells specifically killed CD30^+^ tumor cells *in vitro* with efficiency similar to that of murine HRS3-CAR-T, and these T cells significantly inhibited the established CD30^+^ human lymphoma tumor cells in immunodeficient mice. These findings indicate that hHRS3-CAR-T cells are a promising cellular therapeutic agent and warrants further clinical exploration.

## Data Availability

The original contributions presented in the study are included in the article/Supplementary Material, further inquiries can be directed to the corresponding authors.

## Abbreviations

AFM13, HRS3-based bispecific antibody; ALCL, anaplastic large cell lymphoma; CARs, chimeric antigen receptors; CAR-T, chimeric antigen receptor T-cells; CD19, cluster of differentiation 19; hHRS3-CAR, humanized HRS3 scFv-based CAR; CFSE, carboxyfluorescein succinimidyl ester; HL, Hodgkin’s lymphoma; HRS3, murine anti-CD30 monoclonal antibody; HRS3-CAR, murine HRS3 scFv-based CAR; PI, Propidium iodide; scFv, single chain antibody fragments; Tcm, central memory cells; Tem, effector memory cells; TNFRSF8, tumor necrosis factor receptor superfamily member 8.
