# Supplementary material for: Humanized CD30-Targeted Chimeric Antigen Receptor T Cells Exhibit Potent Preclinical Activity Against Hodgkin’s Lymphoma Cells
Source: Front Cell Dev Biol. 2022 Jan 12;9:775599. doi: 10.3389/fcell.2021.775599 (PMC8790321; doi:10.3389/fcell.2021.775599)
Supplement: Supplementary file 1 [file DataSheet1.docx]

**
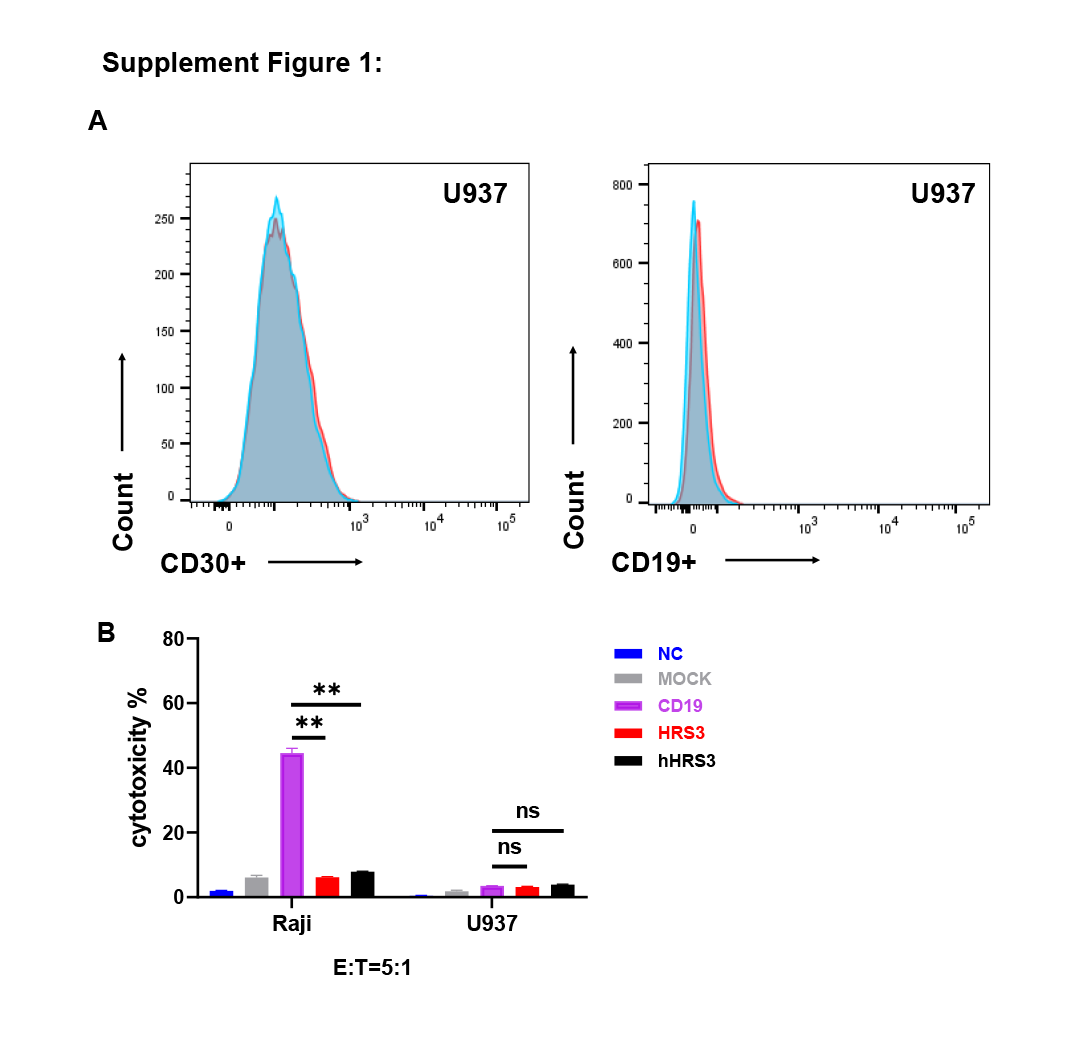
Supplement figure 1:**

(A) CD30 and CD19 expression in U937 cells assessed by flow cytometry. (B) Cytotoxicity of hHRS3-CAR-T, HRS3-CAR-T and control group toward U937 cells. The data were derived from different donor cells and the in vitro experiments were repeated independently at least three times.


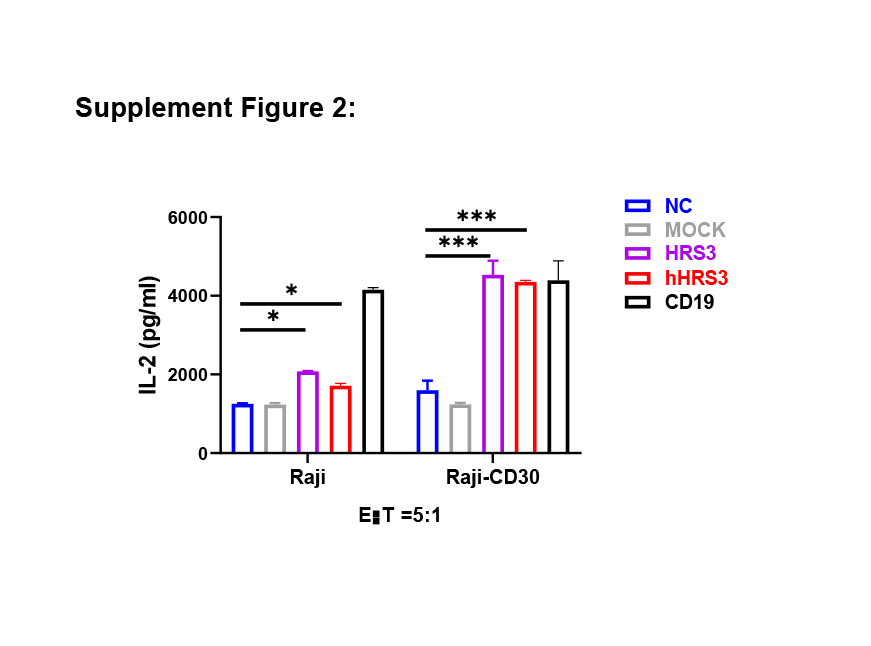


**Supplement figure 2:**

Cytokine IL-2 content in the supernatant of cells from different treatment groups (containing uninfected T cells) co-cultured with Raji, Raji-CD30 cells for 24 hours. The data were derived from different donor cells and the in vitro experiments were repeated independently at least three times. The results are presented as the mean volume ± SD, * p-value < 0.05, ** p-value < 0.01, *** p-value < 0.001 vs NC.


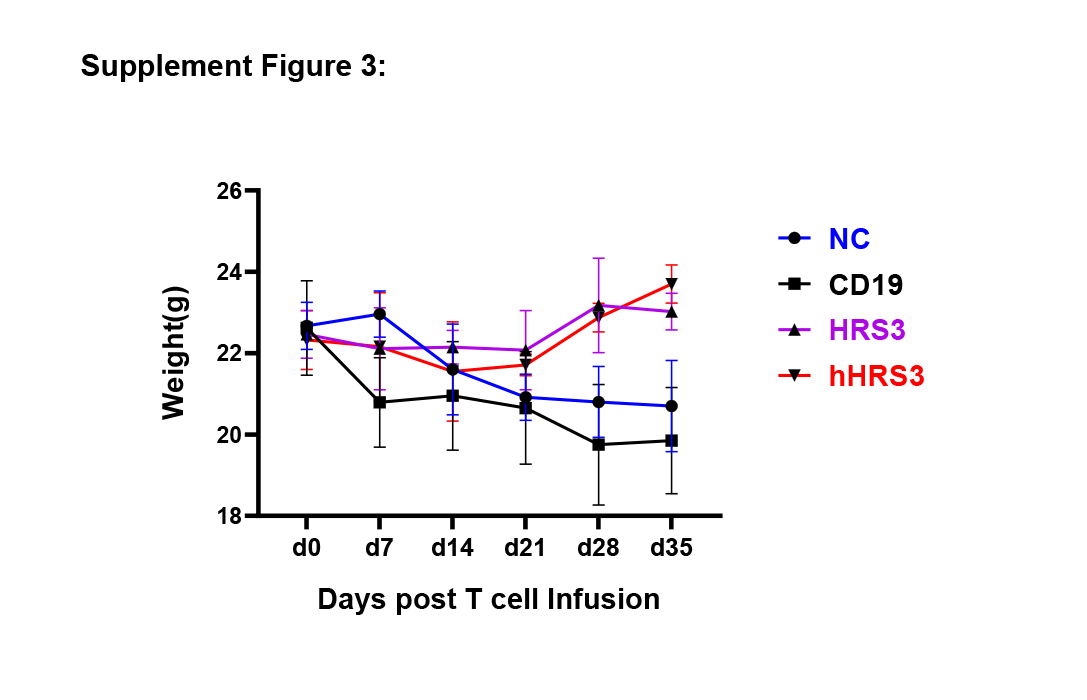


**Supplement figure 3:**

Quantification of body weight mice for each group. Body weights of mice presented as the mean ± standard deviation of 6 mice in each group. Each experiment included 6 mice per group and was repeated twice (total n = 12 mice per group). Data are representative of two independent experiments.
